# Digital health, digital medicine, and digital therapeutics in cardiology: current evidence and future perspective in Japan

**DOI:** 10.1038/s41440-023-01317-8

**Published:** 2023-05-31

**Authors:** Akihiro Nomura

**Affiliations:** 1 College of Transdisciplinary Sciences for Innovation, Kanazawa University, Kanazawa, Japan; 2 Department of Cardiovascular Medicine, Kanazawa University Graduate School of Medical Sciences, Kanazawa, Japan; 3 Frontier Institute of Tourism Sciences, Kanazawa University, Kanazawa, Japan; 4 Department of Biomedical Informatics, CureApp Institute, Karuizawa, Japan

**Keywords:** Digital health, Digital medicine, Digital therapeutics, mHealth

## Abstract

Ten years passed since Japan set out the Action Plan of Growth Strategy that declared the initiatives of digitalization for medicine, nursing care, and healthcare to achieve the world’s most advanced medical care. The initiatives formed the foundation of the Japanese national strategy and have been continuously refined, resulting in the current environment of digital health and digital medicine. Digital health–related terminologies are organized, such as “digital health,” “digital medicine,” and “digital therapeutics” (DTx), as well as several common digital technologies, including artificial intelligence, machine learning, and mobile health (mHealth). DTx is included in mHealth and is a novel disease treatment option. Also, this article thoroughly describes DTx in Japan and compares it with those in the US and Germany, the leading countries in digital health–related policies, regulations, and their development status. In Japan, two of three DTx applications that have been approved and reimbursed by the Ministry of Health, Labor, and Welfare are explained in detail in relation to cardiovascular medicine. When added to a standard smoking cessation program, the DTx system for nicotine dependence significantly improved the continuous abstinence rate. Moreover, the DTx for hypertension together with the guideline-based hypertension management was effective in patients aged 65 years or younger who were diagnosed with essential hypertension without antihypertensive agents, and it was also found to be cost-effective. DTx in cardiovascular medicine, with consideration on safety, efficacy, and cost-effectiveness, could be widely used not only through basic experiments and clinical studies but also through social implementation.

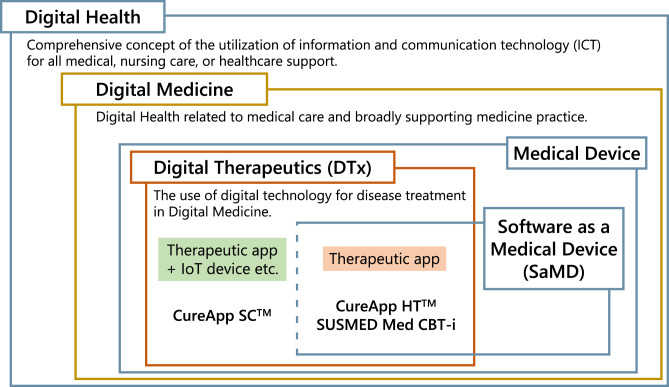

## Introduction

Almost 10 years ago, Japan set out the Action Plan of Growth Strategy that declared the initiatives of digitalization for medicine, nursing care, and healthcare to achieve the world’s most advanced medical care. This action promoted a major push for digital health and digital medicine in Japan. Specific plans included the following: (1) construction of a digital infrastructure for medicine, nursing care, and healthcare; (2) utilization of the digital infrastructure; (3) advanced digitalization of on-site operations; and (4) system establishment for utilizing medical and personal information. These initiatives formed the foundation of the Japanese national strategy and have been continuously refined, resulting in the current environment of digital health and digital medicine [1].

**Fig. 1:**
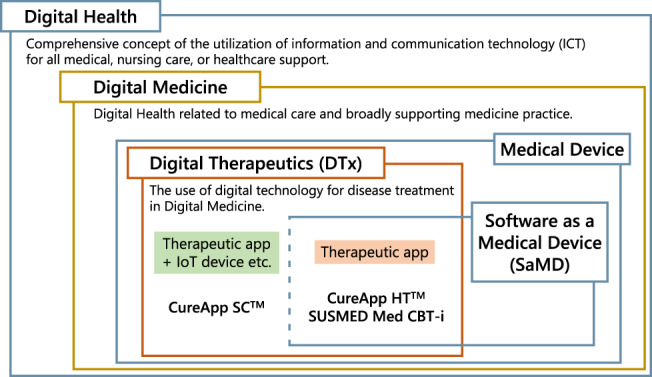
Correlation diagram among digital health-related terminologies

In this article, we define digital health-related terminologies. First, “digital health” is a comprehensive concept of the utilization of information and communication technology (ICT) for all medical, nursing care, or healthcare support. ICT includes digital technologies such as medical big data of genomic and electronic health information, artificial intelligence (AI), or extended reality (XR) [2]. This term often implies the use of the latest and state-of-the-art digital technology to solve various problems in healthcare fields as the objective. Digital technology seems well suited in the following fields: patient treatment; health promotion including primary prevention; the conduct and support of clinical research including decentralized clinical trials; medical education; observation and evaluation of the patients’ clinical course; and public health monitoring for the general population or specific disease cohorts. Moreover, “digital medicine” refers to digital health related to medical care and broadly supporting medicine practice.” [3] In digital medicine, the use of digital technology for disease treatment is referred to as “digital therapeutics (DTx)” (Fig. 1) [4].

For years, various studies and clinical applications other than digital technologies have been conducted to solve healthcare issues. However, digital technology has become one of the powerful tools to solve healthcare-related problems in any medical field and has strongly assisted the evolution of digital health, along with the recent leap in ICT development, miniaturization and technical advantages of mobile devices, easy access to vast and organized data and ample computational resources, and establishment of 5th (5 G) or higher-generation mobile communication systems that allow for high-capacity, low-latency, and multiple connections. Typical digital technologies include online medical services, AI and machine learning (AI/ML), Web 3.0 (web3) and blockchain technology, XR including metaverse, electronic health records (EHR) and personal health records (PHR), and mobile health (mHealth).

### Telehealth and telemedicine

As for online medical service, the Ministry of Health, Labour, and Welfare (MHLW) in Japan has provided “the guidance for the appropriate implementation of telemedicine.” [5] In this guidance, telehealth refers to health promotion and medically related activities using ICT equipment [5]. It includes not only telemedicine but also online medical advice, remote healthcare communication, and real-time online consultation between physicians, similar in concept to digital health. In particular, telemedicine is strictly defined as real-time examination, diagnosis, explanation of laboratory results, and treatment between the physician and the patient, using remote communication tools installed on mobile devices or computers [5]. Of note, regardless of whether it is an insurance-covered medical treatment or not, telemedicine based on this guidance is required.

Owing to the recent coronavirus disease 2019 (COVID-19) pandemic, telemedicine has rapidly and mandatorily gained recognition in Japan. After the state of emergency was declared in April 2020, the MHLW has permitted the use of telemedicine from the first online consultation [6, 7]. Since then, the proportion of hospitals or clinics that could provide telemedicine increased to 15.2% in April 2021 [8]. The revision for medical service fee [9] in 2022 that raised several telemedicine fees (but still less than the face-to-face outpatient fees) may help accelerate the widespread use of telemedicine [10]. However, the number of conducting telemedicine in Japan remains considerably lower than that in European and North American countries [11]. Thus, the advantages and challenges of telemedicine should be reconsidered to further promote its use.

### AI/ML

AI has no single definition. The Japanese Society of Artificial Intelligence defined AI as something aimed to perform advanced inference accurately on a large amount of knowledge data [12]. However, the concept of AI is highly diversified and still under discussion. Thus, when using this term, we need to pay attention to what kind of specific AI technology is referred to [13] Currently, rule-based and ML are frequently used AI technology in medical sciences. In addition, the development of computer resources and easy access to medical big data enable us to utilize ML and its subfield, that is, deep learning, for clinical applications.

One of the primary approaches for medical AI implementation today would be to leverage ML, including deep learning or reinforcement learning, as a tool to obtain the target output [13]. In other words, medical AI aims to maximize the performance of the output as “prediction,” “classification,” or “generation” of diseases or data that are currently required in medicine, and numerous efforts are being made to implement it in society. Recently, a large language model of Generative Pre-trained Transformer 3 (GPT-3) with supervised fine-tuning and reinforcement learning from human feedback as InstructGPT [14] and its dialogue-optimized conversation web-console (ChatGPT) [15] attracts huge attention worldwide. Surprisingly, the ChatGPT has already been scored at or near the passing threshold on the United States Medical Licensing Exam [16]. The products applying these natural language processing models have the potential to rapidly penetrate in every aspect of medical fields soon.

### Web3.0/Metaverse/Blockchain

Web3.0 is a next-generation Internet environment utilizing blockchain technology, and the metaverse supports part of this environment. Blockchain technology is a type of database that processes and records transactions using cryptography, directly connecting terminals on information communication networks [17]. It has excellent tamper resistance for efficient monitoring and data management in clinical trials [18]. The term “metaverse” refers to a virtual space where anyone can communicate similar to the real world and engage in economic activities involving money as both fiat currency and cryptocurrency [19]. Metaverse uses cross reality (XR), including virtual reality (VR) as its utilization technology, and XR is becoming noteworthy in the medical field.

### XR

VR is a technology that creates a virtual environment through a computer, stimulating the human senses and making such an environment perceived as “reality.” [20] Currently, VR controls the visual and auditory senses, and it was often defined as an environment where the external “real” world is completely shut off by a full immersive head-mounted display (HMD). Similar concepts include Augmented Reality (AR) and Mixed Reality (MR), which mainly refer to real-time overlaying (for AR) or merging (for MR) of the environment and objects onto the actual reality we perceived using a see-through HMD or smartphones [20]. Clearly distinguishing them is difficult; thus, a comprehensive concept of XR (cross reality or extended reality) emerged. In the medical field, XR has already been used for medical equipment-level surgical support system [21], medical education [22], and XR-based rehabilitation system [23].

### EHR/PHR

EHR is a collection of electronic medical records stored in an electronic chart originally intended to use only in each hospital or clinic but made shareable and accessible in a specific region or nationwide [24]. EHR contains sensitive personal information; thus, it has been managed mainly by medical institutions. Conversely, the PHR refers to securely usable online medical, health, care and well-being information collected and managed by the person who is being described in the record [25] In PHR, health-related information can be shared and aggregated at the individual level. Thus, even if people visited multiple clinics, PHR can manage not only their medical records but also their lifelong data obtained by wearable devices during daily life.

### mHealth

The term “mHealth” generally refers to digital health using mobile devices. Currently, the most used mobile devices are smartphones and wearable devices. With the advancement of “smart” devices, mobile devices can now measure and estimate not only steps or pulse rates but also electrocardiograms, skin temperature, blood oxygen levels, stress levels, blood pressure, or plasma glucose levels [26]. The wearable devices can also be linked with smartphones to allow viewing, verifying, and processing of biometric data in detail and sharing of data with healthcare providers as needed.

In mHealth, DTx is attracting attention as a novel third option for disease treatment; it is one of the three core treatment pillars, namely, medical, surgical, and digital therapies. I especially focus on DTx in the following chapters.

## What is DTx?

DTx is a novel therapeutic option that provides treatment for illnesses through software applications (apps) delivered via digital devices [4], and is expanding its scope to disease prevention and management. Currently, smartphones and VR HMDs are prevalently used for this purpose. This concept of DTx was introduced in Japan with the revision of the Act on Securing Quality, Efficacy, and Safety of Products Including Pharmaceuticals and Medical Devices, which demonstrated that software programs (i.e., Software as a Medical Device [SaMD]), including standalone apps themselves, could be certified as medical devices (Fig. 1) [27]. Currently, the software app that provides DTx is called “therapeutic apps.” The term “prescription digital therapeutics” is also used, considering that physicians or healthcare professionals “prescribe” the therapeutic app to the patients, let them install it on their digital device, and provide the intended treatment [4]. DTx not only aims to treat illnesses but also provides a direct digital intervention to patients that have a scientifically proven treatment effect, and it has been approved by regulatory agencies. In addition, compared with medical care provided in hospitals or clinics, DTx can provide seamless treatment interventions through mobile digital devices even in patients’ daily lives.

## DTx in the US and Germany

Presently, the US and Germany are particularly leading the digital health-related policies, regulations, and their development status. In the US, regulation of mobile medical applications (MMAs), including DTx, was first issued by the Food and Drug Administration (FDA) in 2013, and was updated in 2015, 2019, and 2022 [28]. Similar to traditional medical devices, MMAs that have a significant impact on patients or medical decision-making requires appropriate regulation processes, including clinical trials. However, the conventional medical device approval process does not automatically adopt the rapidly evolving digital technology used in software or MMA development. Therefore, in July 2017, the FDA launched the Digital Health Innovation Action Plan, which includes the Software Pre-Cert Pilot Program, a system used for faster and safer review and approval processes of digital health products, including MMAs [29]. This innovative system assesses the development capabilities and safety of the development manufacturers rather than each individual medical device software, allowing the companies to bring their FDA-cleared software to market faster and more efficiently. Although the pilot program was completed in September 2022 [30], the FDA continues to develop policies with the Digital Health Center of Excellence, a digital health resource center, to improve regulatory processes related to medical device software, enabling digital health stakeholders to advance all aspects of healthcare through high-quality digital health innovation [31].

Germany is releasing more DTx medical device software to the market than the US. Germany installs the same public health insurance system as Japan, and the implemented policies provide important hints on how to generalize DTx and medical device software. In November 2019, Germany launched the Digital Healthcare Act (*Digitale-Versorgung-Gesetz* or DVG) [32], which explains the review and approval process of low-risk medical devices essentially based on digital technologies such as Digital Health Applications (*Digitale Gesundheitsanwendungen* or DiGA) [33]. Same as FDA, the German Federal Institute for Drug and Medical Devices (*Bundesinstitut für Arzneimittel und Medizinprodukte* or BFArM) assesses DiGA according to the following requirements: safety, functionality, quality, data protection, data security, and positive effects on care under the DVG. However, the DVG’s striking point is that even the DiGA and its manufacturer satisfy all requirements excluding the “positive effects on care,” the DiGA can still provisionally be registered in the BFArM directory [34]. Therefore, even if the manufacturer has not submitted the DiGA’s clinical efficacy validation data through regulation processes, such as clinical trials, the DiGA can still be registered and tentatively reimbursed by health insurance as long as the app’s safety, functionality, quality, data protection, and data security are satisfactory. The provisional reimbursement period is limited to 12 months (or can be extended to 24 months in a specific situation) until the clinical efficacy evaluation is confirmed. However, during this period, the manufacturer can conduct the DiGA’s pivotal trials, or real-world data can be collected while distributing the app in the market with health insurance coverage [34]. As of February 2023, 48 DiGAs have been registered in the BFArM directory. Of these apps, 16 (33%) have reached permanent reimbursement, 5 (approximately 10%) were removed from the list, and 27 (56%) are in the provisional reimbursement period and are closely monitored if they can demonstrate sufficient clinical efficacy to obtain permanent reimbursement [35].

## DTx in Japan

DTx in Japan has been led mainly by several start-ups since 2014 when the Act on Securing Quality, Efficacy, and Safety of Products Including Pharmaceuticals and Medical Devices were revised. Same in the US or Germany, Japan’s medical device software showing a therapeutic effect for diseases needs to be regulated by the MHLW. In 2020, to further promote early implementation of novel SaMD products, including DTx apps in Japan, the MHLW launched the Digital Transformation Action Strategies in Healthcare (DASH) for SaMD [36]. This strategy included the following: (1) seeking promising technologies; (2) arranging and disclosing the concept of a review process specialized in SaMD; (3) centralizing the SaMD consultation service; (4) establishing a SaMD-compatible rapid, efficient, and flexible review system; and (5) reinforcing the review system for early SaMD implementation. Such regulatory efforts to implement SaMD have improved the related guidance and guidelines [37], leading to a better environment to develop medical device software in Japan.

As of February 2023, two types of DTx (for nicotine addiction [CureApp SC^TM^] and for hypertension [CureApp HT^TM^]) have been approved and reimbursed by the MHLW in Japan. Additionally, DTx for insomnia (SUSMED Med CBT-i) has newly been cleared by MHLW [38]. The following section focuses on the two former DTx apps relating to cardiovascular medicine.

### DTx system for nicotine dependence

The CureApp SC^TM^ DTx for nicotine dependence is a therapeutics system that aims to provide intervention and support for psychological dependence to quit smoking in addition to the 12-week standard smoking cessation program in Japan [39]. This DTx system consists of a smartphone therapeutic app, a Bluetooth-paired mobile checker device for exhaled carbon monoxide (CO), and a web-based personal computer software for physicians [40]. It provides individually tailored behavioral therapy and quit-smoking guidance content through a therapeutic app, thereby intensifying the treatment for psychological dependence on smoking. Moreover, an equipped mobile CO breath analyzer allows patients to measure their expiratory CO levels daily and view their cessation progress through a smartphone app or web-based software for physicians. A multicenter randomized controlled trial assessed the usefulness of the DTx for nicotine dependence [41]. A total of 584 patients diagnosed with nicotine dependence were allocated to either of the following groups: intervention group (using the DTx system for nicotine dependence in addition to a standard smoking cessation program) and control group (using a sham app in addition to a standard smoking cessation program). The primary outcome of the continuous abstinence rate from weeks 9 to 24 was significantly higher in the DTx intervention group than in the control group (63.9% vs. 50.5%; odds ratio [OR], 1.73; 95% confidence interval [CI], 1.24–2.42, *P* = 0.001), and this DTx add-on effect continued at least up to 52 weeks. Hence, the DTx system for nicotine dependence significantly improved the continuous abstinence rate when added to a standard smoking cessation program. Based on these results, the CureApp SC^TM^ DTx system was approved and reimbursed by the MHLW in Japan in 2020 as the first DTx in Asia.

### SaMD DTx app for hypertension

The CureApp HT^TM^ DTx for hypertension is a SaMD therapeutic app that aims to provide continuous treatment for high blood pressure, not only during intermittent clinic visits but also in their daily life. This app was developed to efficiently support and maximize the blood pressure-lowering effect of lifestyle modification [42], which is recommended for all patients with high blood pressure by the hypertension management guidelines [43, 44]. Although many physicians think that hypertension treatment links directly to pharmacological therapy, nonpharmacological therapy has also demonstrated robust blood pressure-lowering effects. Nonpharmacological therapy includes a low-salt diet, weight reduction, regular exercise, moderate alcohol consumption, good sleep, stress management [45, 46]. With this background in the algorithm, the DTx app for hypertension aims to educate, practice, and habituate each nonpharmacological therapy for patients with hypertension through the app during daily life outside hospitals or clinics. The app first provides knowledge and techniques to the users for the six non-pharmacological therapy for hypertension (Step 1: input and education). Next, with the app’s support, the users implement specific lifestyle modifications related to the nonpharmacological therapy based on the knowledge and techniques obtained in Step 1 (Step 2: app-supported experiences). Finally, the users independently set, implement, and evaluate their own goals and achievements of lifestyle modification and truly habituate the target nonpharmacological therapy in their daily life (Step 3: self-planning and evaluation) [47].

The efficacy of DTx for hypertension was tested in the HERB-DH1 pivotal clinical trial [48]. The trial enrolled 390 patients aged 65 years or younger who had essential hypertension (grade I or II) but were not taking antihypertensive agents; they were then allocated to either of the DTx intervention group (received the DTx app for hypertension and lifestyle modification guidance according to the guidelines) or the control group (only received lifestyle modification education according to the guidelines) [47]. The primary endpoint of the change in 24-hour systolic blood pressure by ambulatory blood pressure monitoring from baseline (week 0) to week 12 was −4.9 and −2.3 mmHg mmHg in the DTx intervention and control groups, respectively. Hence, the DTx app intervention group had a significantly greater reduction in blood pressure than the control group (mean difference, −2.4 mmHg; 95% CI, −4.5 to −0.3; *P* = 0.024). Additionally, the reduction of morning home systolic blood pressure from baseline to week 12 was greater in the DTx intervention group than in the control group (−10.6 mmHg vs. −6.2 mmHg; mean difference, −4.3 mmHg; 95% CI, −6.7 to −1.9; *P* < 0.001). Furthermore, these blood pressure reduction effects persisted at week 24 at least. In conclusion, the DTx for hypertension in addition to the guideline-based hypertension management was effective in patients aged 65 years or younger who had essential hypertension without antihypertensive agents.

On top of that, we conducted a cost-effectiveness analysis of the DTx for hypertension by using the background characteristics and effect data of both intervention and control groups in the HERB-DH1 trial [49]. In this analysis, we examined the medical economic effects of using the therapeutic app of DTx for hypertension with a time horizon. The differences in medical costs and quality-adjusted life years (QALY) between the DTx intervention group and the control group were 110 717 yen (higher in the DTx intervention group) and 0.092 (longer in the app intervention group). Therefore, the incremental cost-effectiveness ratio (ICER) was calculated to be 1 199 880 yen/QALY [49]. This ICER value was lower than the “willingness-to-pay” threshold of 5 million yen/QALY, which is one of the acceptable medical costs for each increase in 1 QALY. Thus, prescribing the DTx app might be cost-effective through life. Considering these series of evidence, the CureApp HT^TM^ DTx for hypertension was cleared and was reimbursed by the MLHW in Japan as the world’s first hypertension therapeutic app in 2022.

## Conclusions and future perspective

This review introduces the latest and various digital health technologies with specific terminologies along with the DTx in cardiovascular medicine. Although only three DTx apps have been approved by MHLW in Japan, several manufacturers, including DTx start-ups and pharmaceutical companies, continuously develop DTx and conduct clinical research to obtain regulatory approval. The number of DTx development pipelines in Japan surpasses more than 30, which continues to increase every year (Table 1). The movement to promote DTx in cardiovascular medicine, which applies various digital technologies to patients with cardiovascular diseases and considers the technologies’ safety, efficacy, and cost-effectiveness, will accelerate not only through basic experiments and clinical studies but also through social implementation.

**Table 1:** Summary of DTx development status in Japan as of February 2023

Target indication	Manufacturer	Product name	Development stage	Partner	Reimbursement	Comment
**Neuropsychological**						
Tobacco use disorder	CureApp	CureApp SC	Commercial	Keio University	✓	TA+IoT device
Insomnia	SUSMED	Med CBT-i	MHLW cleared	Kurume University	ongoing	TA
ADHD	Akilli/Shionogi	SDT-001	Pivotal	-	-	Tablet FDA cleared
Alcohol use disorder	CureApp	ALMIGHT	Proof of concept	Kurihama medical and addiction center	-	TA
Depression	BiPSEE/Meiji Seika	Rumination	Proof of concept	Kochi University	-	VR
Depression	Tanabe-Mitsubishi	MTD-810	Proof of concept	Kyoto University National Center of Neurology and Psychiatry	-	TA
Stressor-related disorders	Logos Science	Dr.appli	Proof of concept	Waseda University	-	TA
Postpartum depression	Social Service	-	Discovery	-	-	TA
Depression	Jolly Good/Teijin	-	Discovery	-	-	VR
Schizophrenia	Jolly Good/Otsuka	-	Discovery	-	-	VR
Persistant complex bereavement disorder	SUSMED	-	Discovery	University of Zurich	-	TA
Obsessive compulsive disorder	emol	-	Discovery	Hyogo Medical University	-	TA
**Cardiometabolic**						
Hypertension	CureApp	CureApp HT	Commercial	Jichi Medical University	✓	TA
Chronic renal disease	SUSMED	-	Proof of concept	Tohoku University	-	TA
Diabetes	WellDoc/Astellas	BlueStar	Proof of concept	-	-	TA, FDA cleared CE mark
Diabetes	Asken	Asken	Proof of concept	Kyoto University	-	TA
CHF/Cardiac rehabilitation	CureApp	-	Discovery	Yumino Heart Clinic	-	TA
Cardiac rehabilitation	CaTe	-	Discovery	-	-	TA
Diabetes	MICIN/Terumo	-	Discovery	-	-	TA
**Gastrointestinal**						
Nonalcoholic steatohepatitis	CureApp	NASH app	Pivotal	The University of Tokyo	-	TA
Irritable bowel syndrome	MICIN	-	Discovery	-	-	TA
**Cancer**						
Breast cancer	SUSMED	-	Proof of concept	National Cancer Center Japan	-	TA
Advance care planning	SUSMED	-	Proof of concept	National Cancer Center Japan	-	TA
Cancer support	CureApp/Dai-ichi Sankyo	-	Discovery	-	-	TA
Postmastectomy pain syndrome	SUSMED	-	Discovery	Nagoya City University	-	TA
Opioid induced constipation	SUSMED	-	Discovery	National Cancer Center East	-	TA
**Others**						
Chronic pain	SUSMED	-	Discovery	Nagoya City University	-	TA
Chronic back pain	CureApp	-	Discovery	Fukushima Medical University	-	TA
Migrane	Hedgehog MedTech	-	Discovery	-	-	TA
Otolaryngology	SUSMED/KYORIN	-	Discovery	-	-	TA
Amblyopia	Innojin/Sumitomo	-	Discovery	-	-	VR

*ADHD* Attention-deficit hyperactivity disorder, *CE* European Conformity, *CHF* Chronic Heart failure, *FDA* Food and Drug Administration, *IoT* internet of things, *TA* therapeutic app, *VR* virtual reality
